# *De Novo* Transcriptome of *Brassica juncea* Seed Coat and Identification of Genes for the Biosynthesis of Flavonoids

**DOI:** 10.1371/journal.pone.0071110

**Published:** 2013-08-19

**Authors:** Xianjun Liu, Ying Lu, Yuhui Yuan, Shuyan Liu, Chunyun Guan, Sheyuan Chen, Zhongsong Liu

**Affiliations:** Oilseed Crops Institute, Hunan Agricultural University, Changsha, Hunan, China; Nanjing Agricultural University, China

## Abstract

*Brassica juncea*, a worldwide cultivated crop plant, produces seeds of different colors. Seed pigmentation is due to the deposition in endothelial cells of proanthocyanidins (PAs), end products from a branch of flavonoid biosynthetic pathway. To elucidate the gene regulatory network of seed pigmentation in *B. juncea*, transcriptomes in seed coat of a yellow-seeded inbred line and its brown-seeded near- isogenic line were sequenced using the next-generation sequencing platform Illumina/Solexa and *de novo* assembled. Over 116 million high-quality reads were assembled into 69,605 unigenes, of which about 71.5% (49,758 unigenes) were aligned to Nr protein database with a cut-off E-value of 10^−5^. RPKM analysis showed that the brown-seeded testa up-regulated 802 unigenes and down-regulated 502 unigenes as compared to the yellow-seeded one. Biological pathway analysis revealed the involvement of forty six unigenes in flavonoid biosynthesis. The unigenes encoding dihydroflavonol reductase (DFR), leucoantho-cyanidin dioxygenase (LDOX) and anthocyanidin reductase (ANR) for late flavonoid biosynthesis were not expressed at all or at a very low level in the yellow-seeded testa, which implied that these genes for PAs biosynthesis be associated with seed color of *B. juncea*, as confirmed by qRT-PCR analysis of these genes. To our knowledge, it is the first time to sequence the transcriptome of seed coat in *Brassica juncea*. The unigene sequences obtained in this study will not only lay the foundations for insight into the molecular mechanisms underlying seed pigmentation in *B.juncea*, but also provide the basis for further genomics research on this species or its allies.

## Introduction

*Brassica juncea* (L.) Czern & Coss is grown in China, India, Canada, Australia and Russia as an oilseed, condiment or vegetable crop [1], [2], [3]. This species produces black, brown or yellow seed. The varieties distinct in seed color have many differences in agronomic traits such as oil and protein content [4], [5], seed dormancy [6], seed germination [7]. The seed pigments of the *Brassica* species are predominantly secondary flavonoid metabolites, i.e., flavonols and proanthocyanidins (PAs) [8], [9]. Flavonols accumulate in cotyledons and give seeds a yellow cue while PAs, also known as condensed tannins, deposit only in seed coat and bring about browning through oxidation [10]. PAs, oligomers of (−)-epicatechin, are synthesized in the late stage of flavonoid biosynthesis from dihydroflavonols by catalysis of dihydroflavonol 4-reductase (DFR), leucoanthocyanidin reductase (LODX) and anthocyanidin reductase (ANR) [11]. The genes encoding these enzymes and many other genes participating in flavonoid biosynthesis have been cloned from a dozen of plant species including *Arabidopsis* [11], grape [12], soybean [13]. In *Arabidopsis*, mutants of the genes for flavonoid biosynthesis produce *transparent testa* (*tt*) and yellow or pale yellow seed. However, only few genes regulating flavonoid biosynthesis have been found in *Brassica* species, close allies of *Arabidopsis*. For example, in *B. napus*, *DFR* is found to be associated with seed pigmentation by comparison of DFR activity and PA accumulation in seed coat between a yellow-seeded line and its related black-seeded counterpart [14]. In *B. juncea*, *DFR* and *LODX* are confirmed to participate in seed pigmentation by RT-PCR analysis for gene expression in seed coat between a yellow-seeded line and its brown-seeded near-isogenic lines (NILs) [15], [16]. In *B. rapa*, the *TRANSPARENT TESTA GLABRA 1* (*TTG1*) [17] and the *TRANSPARENT TESTA 8* (*TT8*) [18] encoding a WD-40 regulatory protein and a basic Helix-Loop-Helix transcription factor respectively, are suspected to be involved in seed pigmentation. These results suggest that *Brassica* species synthesize PAs and control the final seed color in a manner analogous to *Arabidopsis*. However, the molecular mechanism underlying seed color is not fully understood in *Brassica*. It is also worth noting that the allotetraploid *B. juncea* and *B. napus* and even the mesopolyploid *B. rapa* have much larger and more complex genome than *Arabidopsis*. As such, for a single-copy gene in *Arabidopsis*, there may be multiple copies in *Brassica* species. For instance, *ANR* [19] and *TT16* [20] each has four copies, and *DFR* two copies [14] in *B. napus*. To date, only a few flavonoid biosynthetic genes have been completely cloned from *Brassica* species [21].

Recently, next-generation sequencing (NGS) technologies have emerged as a revolutionary and high throughput approach that accelerates the genome sequencing and gene function research [22], [23]. NGS-based RNA-seq can provide massive sequences with enormous depth and coverage to easily discover novel gene, splice junctions, fusion transcripts and to make a comparison of gene expression between samples or tissues [22], [23]. It has been applied to analysis of transcriptomes of dozens of plant species including *B. napus* [24]. However, RNA-seq has not been used to study *B. juncea* seed coat up to now.

In this study, two cDNA libraries generated from RNA of seed coat of a *Brassica juncea* yellow-seeded inbred line and its brown-seeded near-isogenic line were sequenced using Illumina/Solexa platform and *de novo* assembled into unigenes. The expression level of unigenes was compared between two libraries on the basis of read per exon kilobase per million (RPKM) analysis. 1,304 unigenes were found to be differentially expressed by at least 2-fold. Forty six unigenes were identified for flavonoid biosynthesis by KEGG analysis. The PA biosynthetic genes such as *DFR*s, *LDOX*s and *ANR*s were found to be significantly down-regulated in the yellow-seeded testa as compared with its black-seeded counterpart, as confirmed by qRT-PCR analysis. These results imply the involvement of PA biosynthetic genes in seed pigmentation in *B. juncea*.

## Results and Discussion

### Seed coat transcriptome sequencing and *de novo* assembly

**Figure 1:**
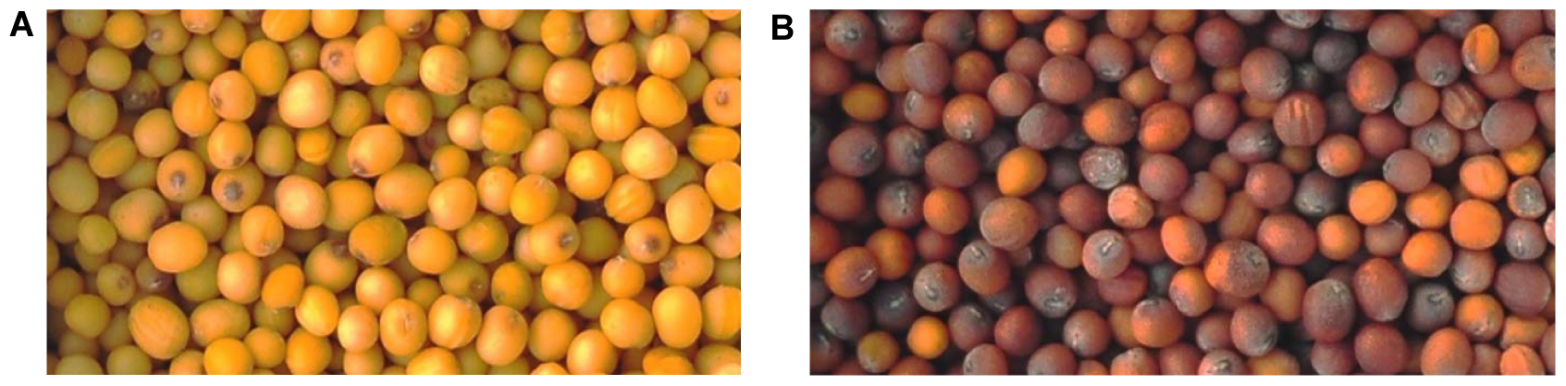
The photographs of mature seeds of *Brassica juncea* accessions used. (A) Sichuan Yellow Seed (SY); (B) The brown-seeded near-isogenic line(NILA) of SY.

**Figure 2:**
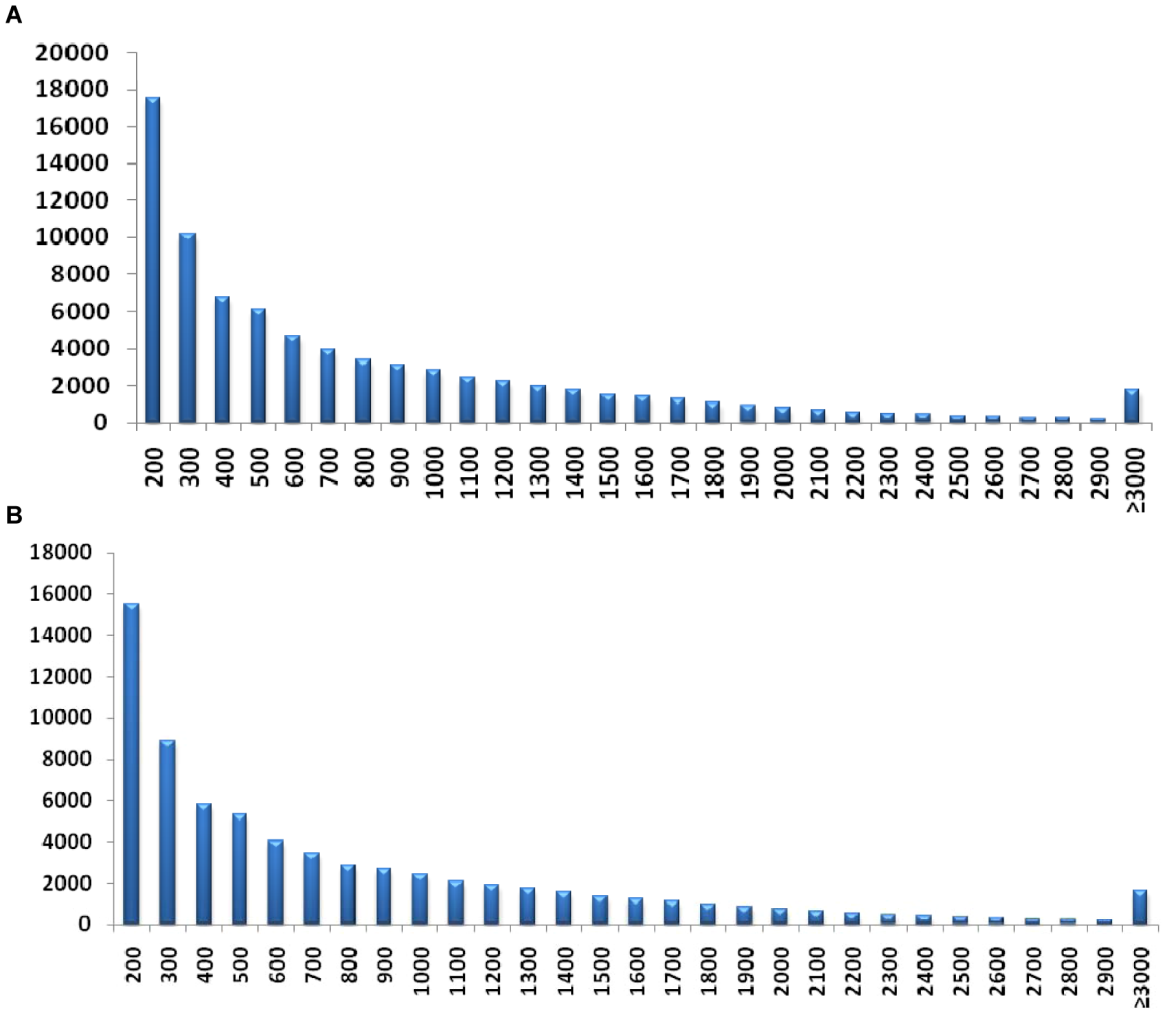
Overview of the *Brassica juncea* seed coat transcriptome assembly. (A) The size distribution of the scaffolds; (B) The size distribution of unigenes.

Total RNA was extracted from seed coat of the *Brassica juncea* yellow-seeded inbred line Sichuan Yellow Seed (SY) and its brown-seeded near-isogenic line A (NILA, BC_8_F_5_) 15 days after pollination (DAP) [25]. The phenotypes of the mature seeds of these lines are shown in Figure 1. A cDNA library was constructed for SY and NILA seed coat, respectively. These cDNA libraries were sequenced by using Illumina HiSeq™ 2000, generating 60,371,476 and 57,465,530 reads of 100-bp in length, respectively. After removing adaptor sequences, low-quality and ambiguous reads, 59,735,444 and 56,423,676 high-quality reads were obtained. An overview of the sequencing and assembly is listed in Table 1. All the raw transcriptome data have been deposited at the sequence read archive (SRA) of the National Center for Biotechnology Information (NCBI). By using the software CLC Genomic Workbench 4.9 (CLC Bio, Denmark), a total of 116,159,120 high-quality reads were assembled into 99,096 contigs, with a minimum contig size of 200 bp, a maximum size of 10,232 bp, an N50 of 1,060 bp and an average contig length of 664 bp. By performing the pair-end joining and gap filling, a total of 79,520 scaffolds were produced, with a minimum scaffold size of 200 bp, a maximum size of 12,040 bp, an N50 of 1,284 bp and an average length of 859 bp. The size distribution of the scaffolds is shown in Figure 2A. By using the software CD-HIT (V.4.5.4) [26], the scaffolds were further assembled into 69,605 unigenes, with a minimum scaffold size of 200 bp, a maximum size of 12,040 bp, an N50 of 1,307 bp and an average length of 868 bp. The size distribution of the unigenes is shown in Figure 2B.

**Table 1:** Summary of sequencing and *de novo* assembling of seed coat transcriptome in *Brassica juncea*.

Item	Library	Number (n)	Sequence (bp)	Average length (bp)	N50 (bp)	Maximum (bp)
Raw read	SY	60,371,476	6,037,147,600	100	-	-
	NILA	57,465,530	5,746,553,000	100	-	-
Clean read	SY	59,735,444	5,537,475,659	92.7	-	-
	NILA	56,423,676	5,320,752,647	94.3	-	-
Total	Contig	99,096	65,776,836	664	1,060	10,232
	Scaffold	79,520	68,319,851	859	1,284	12,040
	Unigene	69,605	60,382,999	868	1,307	12,040

### Functional annotation

**Figure 3:**
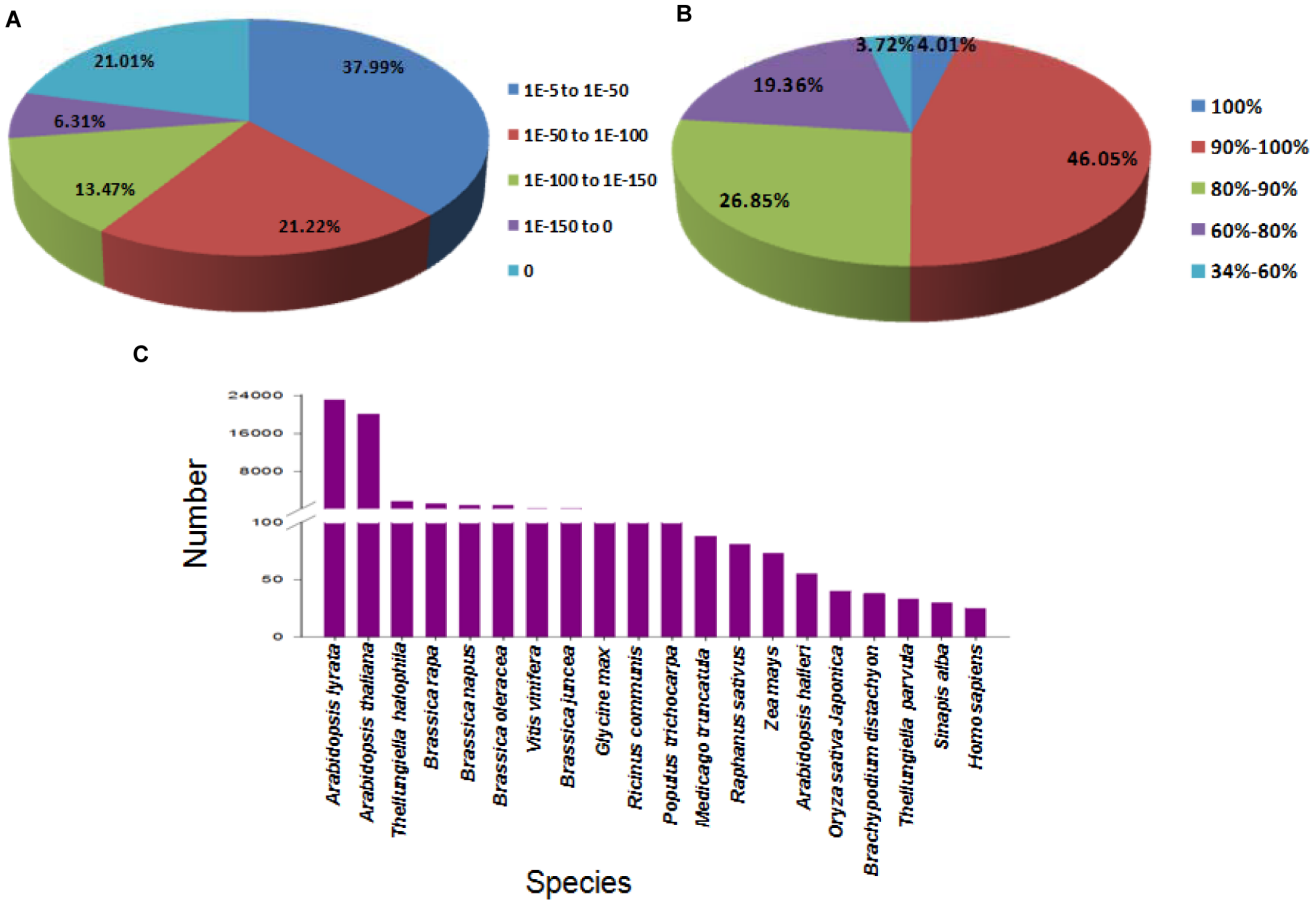
Characteristics of homology search of *Brassica juncea* seed coat unigenes. (A) E-value distribution of the top Blastx hits against the non-redundant (Nr) protein database for each unigene; (B) Similarity distribution of the best Blastx hits for each unigene; (C) Number of unigenes matching the 20 top species using Blastx in the Nr database.

For functional annotation of *B.juncea* seed coat transcriptome, the unigene sequences were blasted against NCBI non redundant (Nr) protein database using a cut-off E-value of 10^−5^. A total of 49,758 unigenes (71.5% of all the assembled unigenes) were aligned to Nr protein database. Among these aligned unigenes, 62.01% had an E-value of less than 1.0E^−50^ and showed very strong homology while the remaining 37.99% had an E-value of between 1.0E^−5^ to 1.0E^−50^ (Figure 3A). The similarity distribution showed that 50.06% of these aligned unigenes had a similarity higher than 90%, 46.21% between 60% and 90% and only 3.72% lower than 60% (Figure 3B). For species distribution, approximately 96.49% of these aligned unigenes were matched with sequences from 6 top-hit species, i.e., *Arabidopsis lyrata* (46.59%), *A. thaliana* (40.59%), *Thellungiella halophila* (3.37%), *B. rapa* (2.38%), *B. napus* (1.87%), and *B. oleracea* (1.69%), which all fall into *Brassicaceae*. The 20 top-hit species based on Nr annotation are shown in Figure 3C.

### Gene Ontology (GO) classification

**Figure 4:**
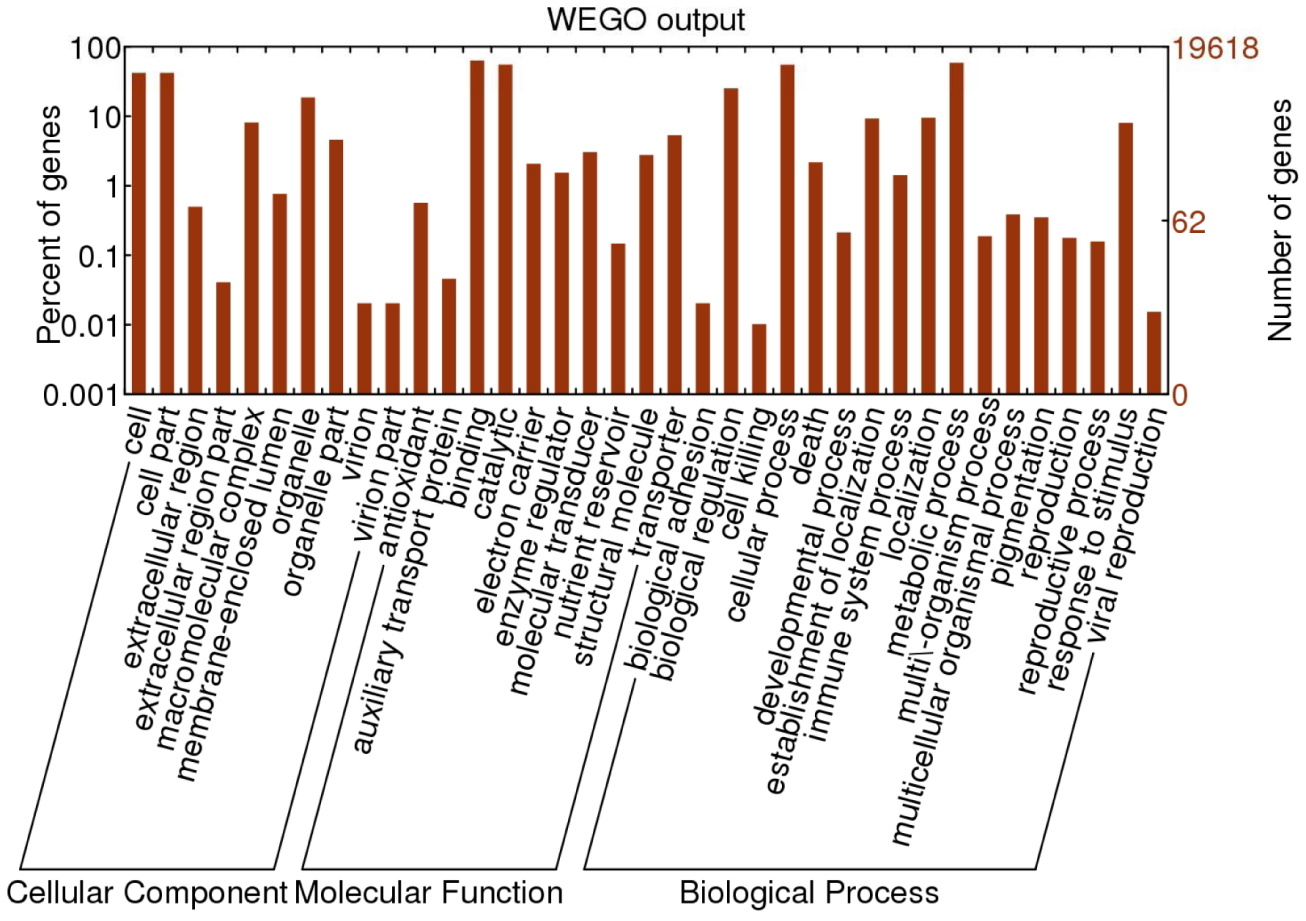
GO classification of unigenes of *B.juncea* seed coat.

GO classification based on sequence homology revealed that 19,618 out of all the assembled unigenes were categorized into 37 functional groups (Figure 4). The three major categories (biological process, cellular component, and molecular function) were assigned 31,026, 22,918 and 26,267 GO terms, respectively (Figure 4). In the ‘biological process’ category, the unigenes related to ‘metabolic processes’ (58.85%) and ‘cellular processes’ (55.05%) were predominant while in the ‘cellular component’ category, ‘cell parts’ (42.05%) and ‘cell’ (42.05%) were found to be the most abundant class. Under the ‘molecular function’ category, the majority of unigenes were involved in ‘binding’ (63.28%) and ‘catalytic activities’ (55.09%).

### COG classification

**Figure 5:**
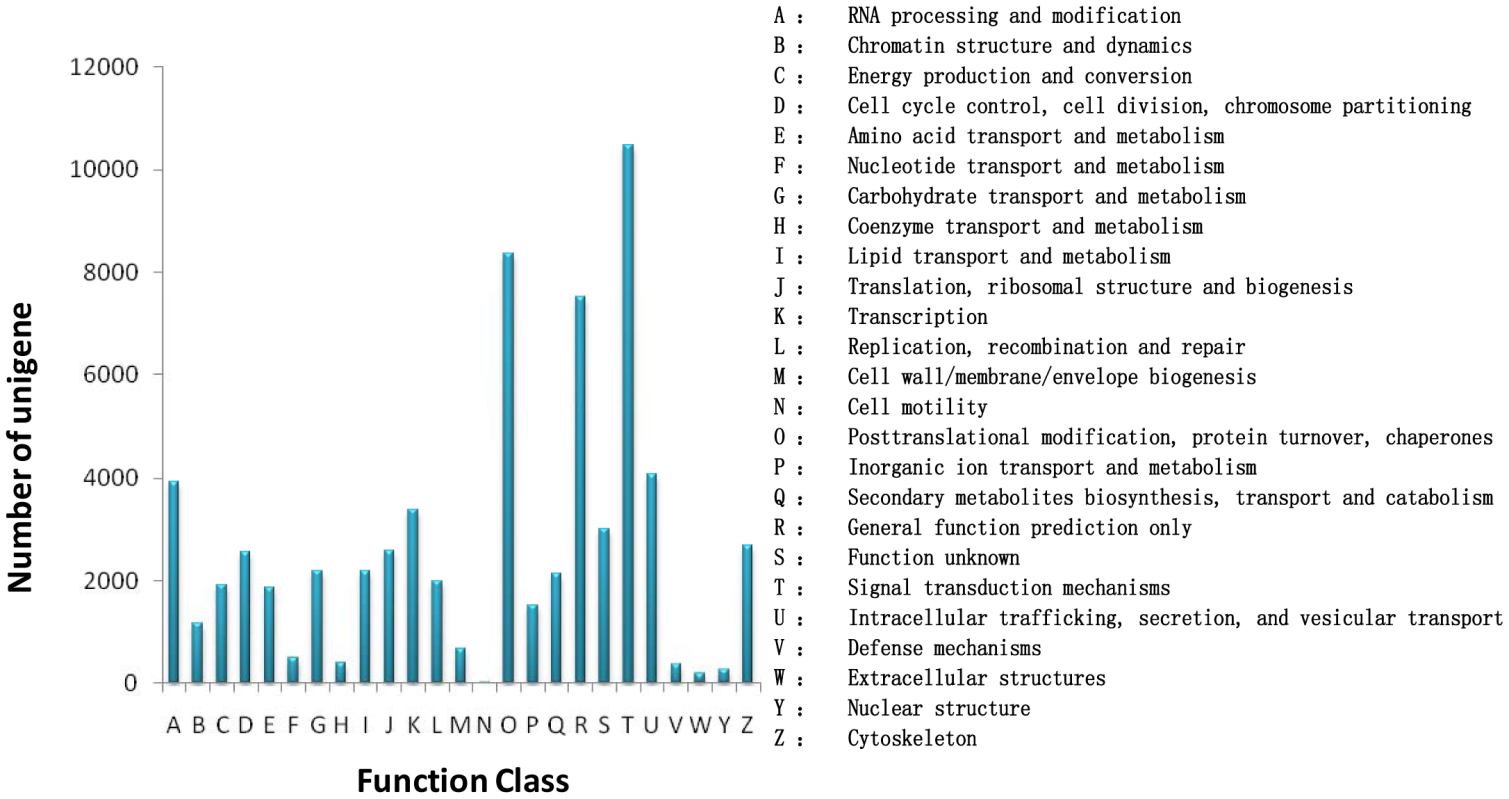
COG function classification of transcriptome.

COG classification of 49,758 Nr hits indicated that 25,140 unigenes were clustered into 25 functional categories (Figure 5). ‘Signal transduction mechanisms’ (10,471 unigenes, accounting for 41.6%) was found to be the major COG category, followed by ‘Posttranslational modification, protein turnover, chaperones’ (8,356, 33.2%), ‘General function prediction only’ (7,519, 29.9%), ‘Intracellular trafficking, secretion, and vesicular transport’ (4,081, 16.2%), ‘RNA processing and modification’ (3,921, 15.6%). ‘Cell motility’ (17, 0.01%) was the smallest group.

### KEGG classification

Analysis of 69,605 unigenes through Kyoto Encyclopedia of Genes and Genomes (KEGG) [27] showed that 14,998 unigenes were assigned to 258 pathways(Table S1). The major pathways containing hundreds of unigenes were metabolic pathways (3,506 unigenes, accounting for 23.37%), biosynthesis of secondary metabolites (1,785, 11.9%), microbial metabolism in diverse environments (802, 5.35%), RNA degradation (538, 3.59%), and ribosome (535, 3.57%). We concentrated on the ‘biosynthesis of secondary metabolites’ pathway in relation to seed pigmentation and found 154 unigenes for ‘Phenylpropanoid biosynthesis’, 114 for ‘Phenylalanine, tyrosine and tryptophan biosynthesis’, 46 for ‘Flavonoid biosynthesis’ and 9 for ‘Flavone and flavonol biosynthesis’ in *B.juncea* seed coat.

### Identification of transcription factors in the seed coat transcriptome

All the assembled unigenes were aligned against the AGRIS (*Arabidopsis* Gene Regulatory Information Server) database by Blastx with *E* value of below 10^−5^ and identity of over 70%. A total of 2,347 unigenes were indentified to belong to forty eight putative transcription factors (TF) families (Table S2). For MYB and bHLH families, two major TF families whose members regulate flavonoid biosynthesis in plants [10], [12], [28], 100 and 190 unigenes were identified, respectively.

### Transcripts differentially expressed between the yellow- and brown-seeded testa

**Figure 6:**
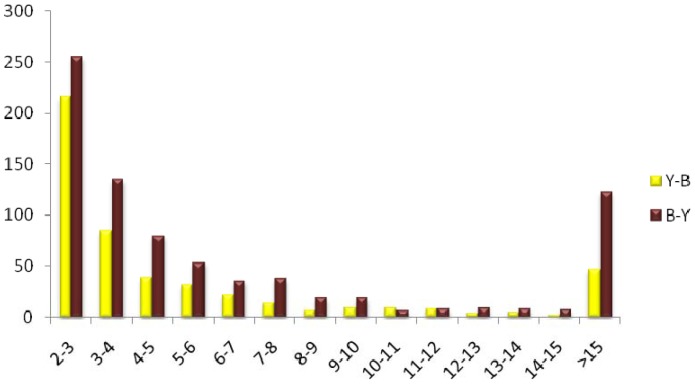
The fold change distribution of differentially expressed between the yellow- and brown-seeded testa of *Brassica juncea*.

**Figure 7:**
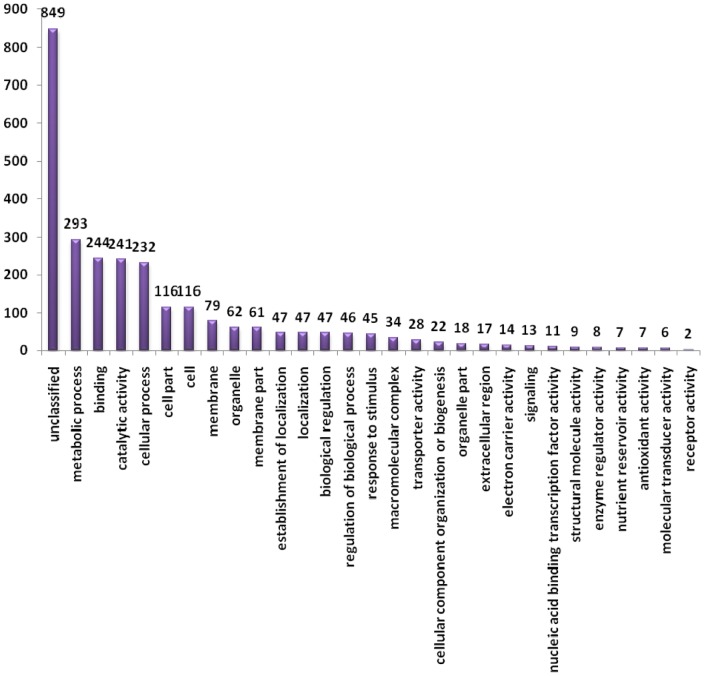
Functional categoried of unigenes differentially expressed between the yellow- and brown-seeded testa of *Brassica juncea*.

To investigate the expression level of unigenes in the yellow- and brown-seeded testa, the number of clean reads was compared between the libraries for each of 69,605 assembled unigenes through RPKM analysis [29]. 1,304 unigenes were found to be differentially expressed between the yellow- and brown-seeded testa, among which 802 unigenes were up-regulated while 502 down-regulated in the brown-seeded testa as compared to the yellow-seeded counterpart (Figure 5; Table S3). Among these unigenes, 170 (12.8%) showed over 15-fold change in expression level as well as 471 (36.4%) showed 2∼3-fold change. The fold change distribution of unigenes differentially expressed between the testae was shown in Figure 6. Annotation of differentially expressed unigenes revealed that 455 unigenes belonged to 28 GO groups while the remaining 849 unigenes could not to be classified (Figure 7).

### Genes from the seed coat transcriptome involved in the flavonoid biosynthetic pathway

**Figure 8:**
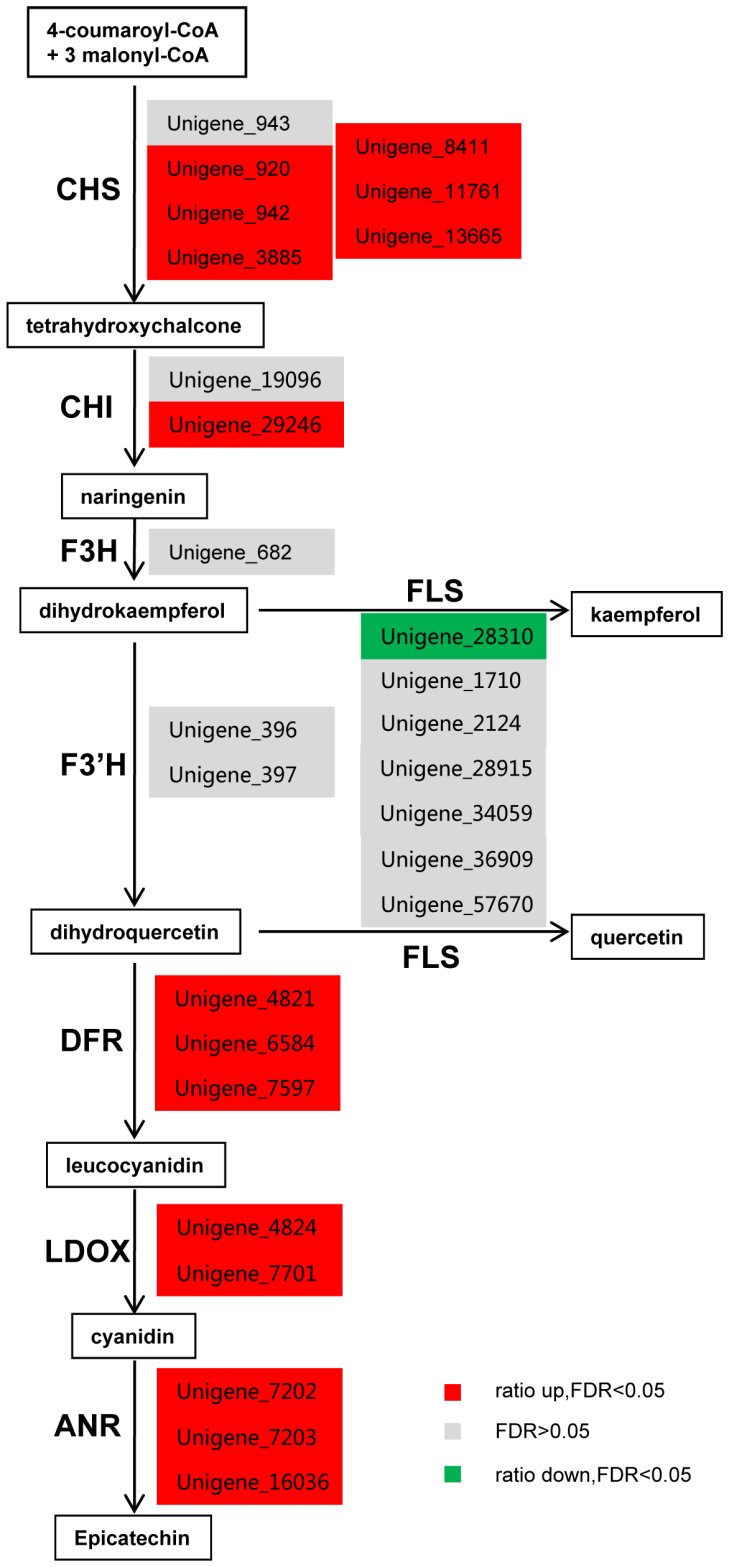
Unigenes involved in the flavonoid biosynthesis pathway in seed coat of *Brassica juncea*. Abbreviation: ANR, anthocyanidin reductase; CHS, chalcone synthase; CHI, chalcone isomerase; DFR, dihydroflavonol 4-reductase; F3H, flavanone 3-hydroxylase; F3′H, flavonoid 3′-hydroxylase; FLS, flavonol synthase; LDOX, leucoanthocyanidin dioxygenase.

In *Arabidopsis*, PA synthesis initiates in the endothelial cells of seed coat at 3 DAP although expression of the genes for PA biosynthesis gradually decreases from 3 DAP onwards [30]. In *B. juncea*, PA accumulation does not occur in seed coat until 10 DAP [31]. Array analysis showed that six flavonoid genes (*chalcone synthase* (*CHS*), *flavonoid 3-hydroxylase* (*F3H*), *flavanol (quercetin) 3-O-methyltransferase* (*FOMT*), *DFR*, *glutathione S-transferase* (*GST*), and *TTG1*) had higher and two (*flavonoid 3′-hydroxylase* (*F3′H*) and *flavonol synthase* (*FLS*)) had lower expression in the developing siliques (22 DAP) of the *B. carinata* brown-seeded accession than in those of the yellow-seeded one [32]. Comparison between secondary-wall-enriched seed coat and developing hypocotyls showed that the transcripts of flavonoid biosynthetic genes *ANR*, *FLS* and *CHS* were more abundant in the middle fraction of seed coat than in the developing hypocotyls of *B. napus* [33]. This mean that flavonoid biosynthetic genes are expressed in seed coat at a higher level, which is consistent with sole deposition of PAs in seed coat [8]. In this study, we used the seed coat for analysis, which eliminates interference from the silique wall or even the embryo. As mentioned above, forty six unigenes were found to be involved in flavonoid biosynthesis of *B. juncea* seed coat. Among them, six unigenes for CHS, and both two unigenes for chalcone isomerase (CHI) were up-regulated and one unigene for FLS down-regulated in the brown-seeded testa as compared to the yellow-seeded one(Figure 8; Table S4). Previous RT-PCR analysis and activity assay of three enzymes DFR, LDOX and ANR for PA biosynthesis have shown that *DFR* and *LDOX* are not expressed in seed coat of *Brassica* yellow seeds [14], [15], [34]. Here, we found no *DFR* unigenes in the yellow-seed testa, but three *DFR* unigenes expressing at a high level in the brown-seeded testa with 10,510, 9,644 and 5,203 reads, respectively (Table S4). Similarly, three *ANR* unigenes were almost not expressed in the yellow-seed testa, although expressed highly in the brown-seeded testa. In addition, two LDOX unigenes were expressed at a much higher level in the brown-seeded testa than in the yellow-seeded one. Taken together, these results suggest that the genes for PA biosynthesis be not expressed or at a very low level and therefore no PAs accumulated in the yellow-seeded testa of *B.juncea*.

### Real-time PCR analysis of the genes involved in the flavonoid biosynthesis pathway

**Figure 9:**
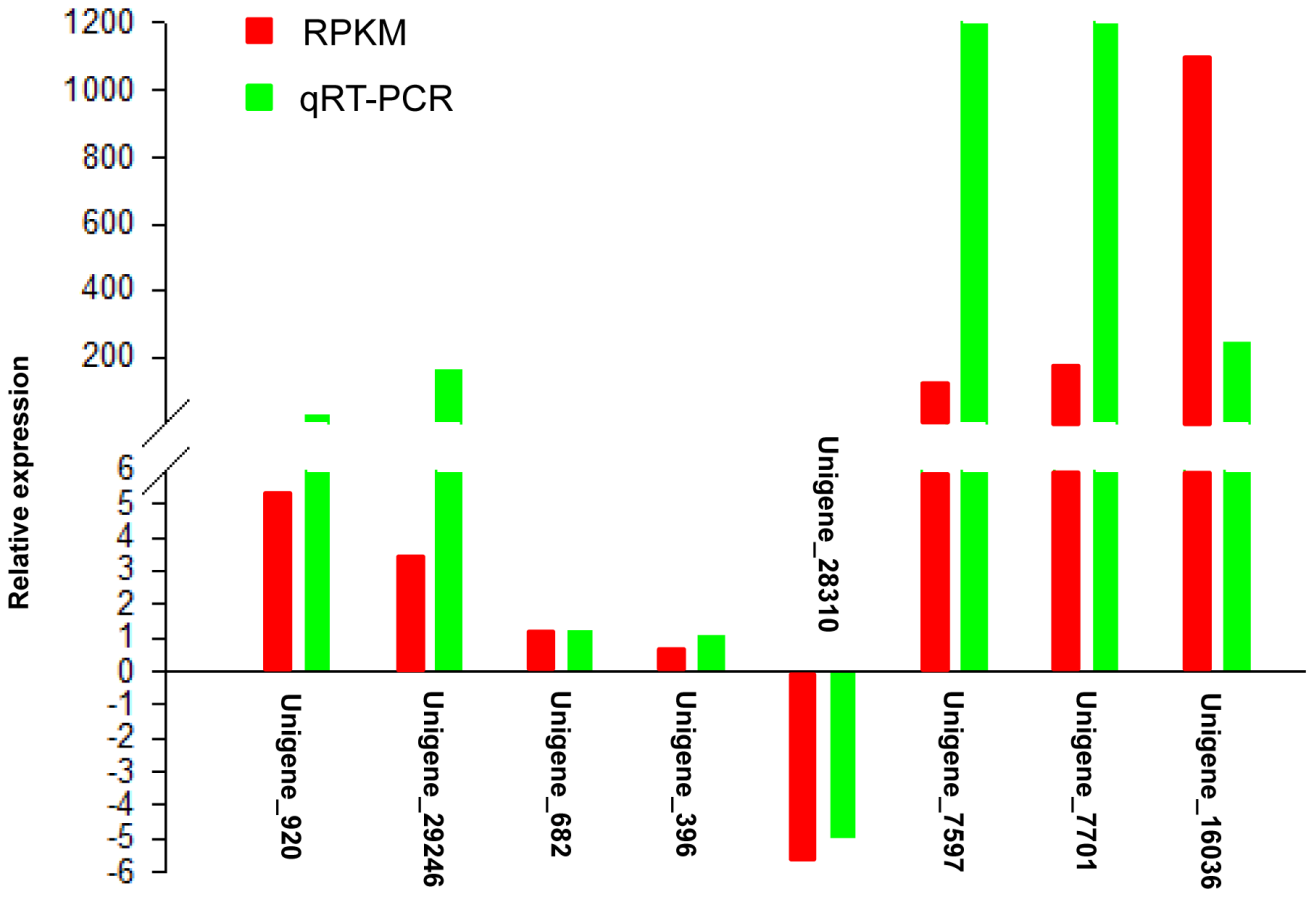
qRT-PCR validation of RPKM analysis of the eight unigenes involved in flavonoid biosynthesis of *Brassica juncea* seed coat.

To confirm the difference in expression level between the accessions found in RPKM analysis, eight unigenes for flavonoid biosynthesis were chosen for qRT-PCR analysis (Figure 8). These unigenes were Unigene_920 (*CHS*), Unigene_29246 (*CHI*), Unigene_7597 (*DFR*), Unigene_7701 (*LDOX*), and Unigene_16036 (*ANR*) which were up-regulated in the brown-seeded testa, Unigene_28310 (*FLS*) down-regulated, Unigene_682 (*F3H*) and Unigene_396 (*F3′H*) unchanged. qRT-PCR data confirmed expression pattern of these unigenes by RPKM analysis (Figure 9, Table S4).

### Conclusion

To the best of our knowledge, this study is the first to apply the Illumina/Solexa platform to investigating the sequences and transcript abundances of genes expressed in seed coats of *Brassica juncea*. This transcriptome analysis has provided a total of 69,605 unigenes among which 71.5% are aligned to Nr database although there is no *Brassica juncea* reference genome sequence of available. Comparison in expression level between the yellow- and the brown-seeded testa totally identified 1,304 differentially expressed unigenes including the key PA biosynthetic genes such as *DFR*s, *LDOX*s and *ANR*s. In sharp contrast to high expression in the brown-seeded testa, expression of the PA biosynthetic genes and accumulation of PAs are inhibited in yellow-seeded testa, which suggests that PAs play a major role in seed pigmentation of *B. juncea*. The unigene sequences obtained in this study will not only lay the foundations for insight into the molecular mechanism underlying seed pigmentation in *B.juncea*, but also provide the basis for further genomics research on this species or its allies.

## Materials and Methods

### Plant materials

A yellow-seed inbred line Sichuan Yellow Seed (SY) and its brown-seeded near-isogenic line A (NILA) (Figure 1) of *Brassica juncea* (L.) Czern & Coss were used in this study. The plants were grown in a greenhouse under a photoperiod of 16 h day and 8 h night at 22°C. Fresh seed coats were excised by hand from the seeds 15 days after pollination, immediately frozen in liquid nitrogen, and stored at −80°C for RNA extraction.

### Construction of cDNA library for sequencing

Total RNA was extracted from seed coats using TRIzol Reagent (Life technologies, US) following the manufacturer's instructions and checked for a RIN number to inspect RNA integration by an Agilent Bioanalyzer 2100 (Agilent technologies, US). Qualified total RNA was purified by RNeasy micro kit (QIAGEN, Germany) and RNase-Free DNase Set (QIAGEN, Germany). Poly(A) mRNA was isolated from qualified total RNA using Oligotex mRNA mini kit(QIAGEN, Germany) and then broken into short fragments which were used as templates for synthesis of the first- and the second-strand cDNA. Two paired-end libraries were synthesized by using the Genomic Sample Prep kit (Illumina, US) per the manufacturer's instructions. Short fragments were purified with the Qubit™ dsDNA HS Assay kit (Invitrogen, US) and connected with different sequencing adapters. Each of the two libraries had an average insert size of 400 bp and was sequenced by Shanghai Biotechnology Corporation (Shanghai, China) using Illumina HiSeq™ 2000. The transcriptome datasets are available at the NCBI Sequence Read Archive (SRA), under the accession number SRA072246.

### *De novo* assembly

Pre-processing and assembling of the raw sequence data were carried out by using CLC Genomics Workbench (version 4.9). This pre-processing included the removal of the adapter sequences, low quality (Q20<20) sequences, shorter-than-15-nucleotide sequences and ambiguous inner regions. For *de novo* assembly, contig scaffolding algorithm was adopted. The parameters were set as follow: length fraction (i.e. minimum length fraction of a read that must match the reference sequence) of 0.5, similarity ratio (i.e. minimum similarity of the match length of a read) of 0.8 and the minimum contig length (minimum length of an assembled contig to be reported) of 200 bp or longer. Then, the scaffolds were clustered by using CD-HIT (V.4.5.4) with 90% similarity cut-off to produce distinct unigenes.

### Functional annotation

Function of unigenes was annotated by Blastx against the NCBI non-redundant (Nr) database or the AGRIS database (http://arabidopsis.med.ohio-state.edu/downloads html) [35] with E-value threshold of 10^−5^. Base on Nr annotation, top-hit species were identified. Gene ontology (GO) classification was obtained by WEGO [36] (http://wego.genomics.org.cn/cgi-bin/wego/index.pl) via GO id annotated by Perl and R program. The unigenes sequences were also aligned to the COG database to predict and classify functions. KEGG pathways were assigned to the unigene sequences using the single-directional best hit (SBH) method on the KEGG Automatic Annotation Server (KAAS) online [37].

### Expression analysis

The RPKM method was used to calculate the unigene expression in this study. The clean reads of each library were mapped to the sequences of each unigene. The significance of difference in gene expression between the yellow- and brown-seed testa was determined by using DEGseq, an R package [38]. False discovery rate (FDR) was applied to identify the threshold of the *P* value in multiple tests [39]. When FDR is less than 0.05 and log_2_ ratio greater than 1 (two-fold change) between the accessions the unigenes were considered as differentially expressed.

### Real-time quantitative RT-PCR(qRT-PCR) analysis

Eight unigenes involved in flavonoid synthesis were chosen for validation by qRT-PCR. The primers designed with the software Primer Premier 5.0 were listed in Table S5. Total RNA was extracted from seed coat tissues 15 DAP using TRIzol RNA extraction method (Tiangen, China) and reverse transcribed into cDNA using PrimeScritH RT reagent kit with gDNA Eraser (Perfect Real Time) (Takara, China). The qRT-PCR was performed with a Bio-Rad CFX-96 RealTime PCR System (Bio-Rad, US) in a final volume of 20 µl containing 2 µl cDNA, 10 µl 2×SYBR premix Ex taqTM (Takara, Japan), 0.4 µl 10 µM the forward and reverse primers each, and 7.2 µl RNase-free water. The thermal cycling conditions were as follows: 95°C 5 min, 45 cycles at 95°C for 5 s for denaturation and 56°C for 25 s for annealing and extension. *TIPS-41* [40] was used as an internal control for normalization to make a comparison of gene expression level between the accessions.

## Supporting Information

Table S1 **Summary of the KEGG pathway and their corresponding gene number in the transcriptome of** ***Brassica juncea*** **seed coat.**
(XLS)

Table S2 **Transcription factors identified in the transcriptome of** ***Brassica juncea*** **seed coat.**
(XLS)

Table S3 **The unigenes differentially expressed between between the yellow- and brown-seeded testa of** ***Brassica juncea*** **.**
(XLS)

Table S4 **The unigenes involved in flavonoid biosynthetic pathway and their coverage in the transcriptome of** ***Brassica juncea*** **seed coat.**
(XLS)

Table S5 **The primers used for analysis of gene expression by qRT-PCR.**
(DOC)

## References

[1] Burton W, Salisbury P, Potts D (2003) The potential of canola quality *Brassica juncea* as an oilseed crop for Australia. Proceedings of the 13th Biennial Australian Research Assembly on Brassicas, Tamworth, NSW, Australia 62–64.

[2] Liu SY, Liu ZS, Guan CY (2007) Advances in germplasm of oilseed *Brassica juncea* . Journal of Plant Genetic Resources 3: 351–358.

[3] Oram R, Kirk J, Veness P, Hurlstone C, Edlington J, et al (2005) Breeding Indian mustard [*Brassica juncea* (L.) Czern.] for cold-pressed, edible oil production-a review. Australian Journal of Agricultural Research 56: 581–596.

[4] Simbaya J, Slominski BA, Rakow G, Campbell LD, Downey RK, et al (1995) Quality characteristics of yellow-seeded *Brassica* seed meals: Protein, carbohydrate, and dietary fiber components. Journal of Agricultural and Food Chemistry 43: 2062–2066.

[5] Shirzadegan M, Röbbelen G (2006) Influence of seed color and hull proportion on quality properties of seeds in *Brassica napus* L. Fette, Seifen, Anstrichmittel 87: 235–237.

[6] Debeaujon I, Léon-Kloosterziel KM, Koornneef M (2000) Influence of the testa on seed dormancy, germination, and longevity in *Arabidopsis* . Plant Physiology 122: 403–414. 10677433 10.1104/pp.122.2.403 PMC58877

[7] Ren C, Bewley JD (1998) Seed development, testa structure and precocious germination of Chinese cabbage (*Brassica rapa* subsp. *pekinensis*). Seed Science Research 8: 385–398.

[8] Auger B, Marnet N, Gautier V, Maia Grondard A, Leprince F, et al (2010) A detailed survey of seed coat flavonoids in developing seeds of *Brassica napus* L. Journal of Agricultural and Food Chemistry 58: 6246–6256. 20429588 10.1021/jf903619v

[9] Fang J, Reichelt M, Hidalgo W, Agnolet S, Schneider B (2012) Tissue-specific distribution of secondary metabolites in Rapeseed (*Brassica napus* L.). PLoS One 7: e48006. 23133539 10.1371/journal.pone.0048006 PMC3485038

[10] Lepiniec L, Debeaujon I, Routaboul JM, Baudry A, Pourcel L, et al (2006) Genetics and biochemistry of seed flavonoids. Annual Review of Plant Biology 57: 405–430. 10.1146/annurev.arplant.57.032905.105252 16669768

[11] Saito K, Yonekura-Sakakibara K, Nakabayashi R, Higashi Y, Yamazaki M, et al (2013) The flavonoid biosynthetic pathway in *Arabidopsis*: structural and genetic diversity. Plant Physiology and Biochemistry In press. 10.1016/j.plaphy.2013.02.001 23473981

[12] Hichri I, Barrieu F, Bogs J, Kappel C, Delrot S, et al (2011) Recent advances in the transcriptional regulation of the flavonoid biosynthetic pathway. Journal of Experimental Botany 62: 2465–2483. 21278228 10.1093/jxb/erq442

[13] Yang K, Jeong N, Moon JK, Lee YH, Lee SH, et al (2010) Genetic analysis of genes controlling natural variation of seed coat and flower colors in soybean. Journal of Heredity 101: 757–768. 20584753 10.1093/jhered/esq078

[14] Akhov LAL, Ashe PAP, Tan YTY, Datla RDR, Selvaraj GSG (2009) Proanthocyanidin biosynthesis in the seed coat of yellow-seeded, canola quality *Brassica napus* YN01-429 is constrained at the committed step catalyzed by dihydroflavonol 4-reductase. Botany 87: 616–625.

[15] Yan ML, Liu XJ, Liu ZS, Guan CY, Yuan MZ, et al (2008) Cloning and expression analysis of Dihydroflavonol 4-Reductase gene in *Brassica juncea* . Acta Agronomica Sinica 34: 1–7.

[16] Yan ML, Liu XJ, Guan CY, Chen XB, Liu ZS (2011) Cloning and expression analysis of an anthocyanidin synthase gene homolog from *Brassica juncea* . Molecular Breeding 28: 313–322.

[17] Zhang J, Lu Y, Yuan Y, Zhang X, Geng J, et al (2009) Map-based cloning and characterization of a gene controlling hairiness and seed coat color traits in *Brassica rapa* . Plant Molecular Biology 69: 553–563. 19039665 10.1007/s11103-008-9437-y

[18] Li X, Chen L, Hong M, Zhang Y, Zu F, et al (2012) A large insertion in bHLH transcription factor *BrTT8* resulting in yellow seed coat in *Brassica rapa* . PLoS One 7: e44145. 22984469 10.1371/journal.pone.0044145 PMC3439492

[19] Nesi N, Lucas MO, Auger B, Baron C, Lécureuil A, et al (2009) The promoter of the Arabidopsis thaliana BAN gene is active in proanthocyanidin-accumulating cells of the *Brassica napus* seed coat. Plant Cell Reports 28: 601–617. 19153740 10.1007/s00299-008-0667-x

[20] Chen G, Deng W, Peng F, Truksa M, Singer S, et al (2013) *Brassica napus TT16* homologs with different genomic origins and expression levels encode proteins that regulate a broad range of endothelium-associated genes at the transcriptional level. Plant Journal 74: 663–677. 23425240 10.1111/tpj.12151

[21] Liu ZS, Sun DH, Liu XJ, Guan CY (2012) Advance in cloning of genes for proanthocyanidin biosynthesis from oilseed *Brassica* species. Journal of Hunan Agricultural University (Natural Sciences) 38: 354–359.

[22] Wang Z, Gerstein M, Snyder M (2009) RNA-Seq: a revolutionary tool for transcriptomics. Nature Reviews Genetics 10: 57–63. 10.1038/nrg2484 PMC2949280 19015660

[23] Van Verk MC, Hickman R, Pieterse CM, Van Wees S (2013) RNA-Seq: revelation of the messengers. Trends in Plant Science 18: 175–179. 23481128 10.1016/j.tplants.2013.02.001

[24] Bancroft I, Morgan C, Fraser F, Higgins J, Wells R, et al (2011) Dissecting the genome of the polyploid crop oilseed rape by transcriptome sequencing. Nature Biotechnology 29: 762–766. 10.1038/nbt.1926 21804563

[25] Liu XJ, Yuan MZ, Guan CY, Chen SY, Liu SY, et al (2009) Inheritance, mapping, and origin of yellow-seeded trait in *Brassica juncea* . Acta Agronomica Sinica 35: 839–847.

[26] Li W, Godzik A (2006) Cd-hit: a fast program for clustering and comparing large sets of protein or nucleotide sequences. Bioinformatics 22: 1658–1659. 16731699 10.1093/bioinformatics/btl158

[27] Kanehisa M, Araki M, Goto S, Hattori M, Hirakawa M, et al (2008) KEGG for linking genomes to life and the environment. Nucleic Acids Research 36: 480–484. 10.1093/nar/gkm882 PMC2238879 18077471

[28] Feller A, Machemer K, Braun EL, Grotewold E (2011) Evolutionary and comparative analysis of MYB and bHLH plant transcription factors. Plant Journal 66: 94–116. 21443626 10.1111/j.1365-313X.2010.04459.x

[29] Mortazavi A, Williams BA, McCue K, Schaeffer L, Wold B (2008) Mapping and quantifying mammalian transcriptomes by RNA-Seq. Nature Methods 5: 621–628. 18516045 10.1038/nmeth.1226 PMC13303166

[30] Dean G, Cao Y, Xiang D, Provart NJ, Ramsay L, et al (2011) Analysis of gene expression patterns during seed coat development in *Arabidopsis* . Molecular Plant 4: 1074–1091. 21653281 10.1093/mp/ssr040

[31] Lu Y, Liu X, Liu S, Yue Y, Guan C, et al (2012) A simple and rapid procedure for identification of seed coat colour at the early developmental stage of *Brassica juncea* and *Brassica napus* seeds. Plant Breeding 131: 176–179.

[32] Li X, Westcott N, Links M, Gruber MY (2010) Seed coat phenolics and the developing silique transcriptome of *Brassica carinata* . Journal of Agricultural and Food Chemistry 58: 10918–10928. 20925379 10.1021/jf102208a

[33] Jiang Y, Deyholos MK (2010) Transcriptome analysis of secondary-wall-enriched seed coat tissues of canola (*Brassica napus* L.). Plant Cell Reports 29: 327–342. 20145934 10.1007/s00299-010-0824-x

[34] Marles M, Gruber MY, Scoles GJ, Muir AD (2003) Pigmentation in the developing seed coat and seedling leaves of *Brassica carinata* is controlled at the dihydroflavonol reductase locus. Phytochemistry 62: 663–672. 12620317 10.1016/s0031-9422(02)00488-0

[35] Yilmaz A, Mejia-Guerra MK, Kurz K, Liang X, Welch L, et al (2011) AGRIS: the Arabidopsis gene regulatory information server, an update. Nucleic Acids Research 39: D1118–D1122. 21059685 10.1093/nar/gkq1120 PMC3013708

[36] Ye J, Fang L, Zheng H, Zhang Y, Chen J, et al (2006) WEGO: a web tool for plotting GO annotations. Nucleic Acids Research 34: W293–W297. 16845012 10.1093/nar/gkl031 PMC1538768

[37] Moriya Y, Itoh M, Okuda S, Yoshizawa AC, Kanehisa M (2007) KAAS: an automatic genome annotation and pathway reconstruction server. Nucleic Acids Research 35: 182–185. 10.1093/nar/gkm321 PMC1933193 17526522

[38] Wang L, Feng Z, Wang X, Wang X, Zhang X (2010) DEGseq: an R package for identifying differentially expressed genes from RNA-seq data. Bioinformatics 26: 136–138. 19855105 10.1093/bioinformatics/btp612

[39] Benjamini Y, Hochberg Y (1995) Controlling the false discovery rate: a practical and powerful approach to multiple testing. Journal of the Royal Statistical Society Series B (Methodological) 57: 289–300.

[40] Chandna R, Augustine R, Bisht NC (2012) Evaluation of candidate reference genes for gene expression normalization in *Brassica juncea* using real time quantitative RT-PCR. PLoS One 7: e36918. 22606308 10.1371/journal.pone.0036918 PMC3350508

