# Supplementary material for: De Novo Transcriptome of Brassica juncea Seed Coat and Identification of Genes for the Biosynthesis of Flavonoids
Source: PLoS One. 2013 Aug 19;8(8):e71110. doi: 10.1371/journal.pone.0071110 (PMC3747200; doi:10.1371/journal.pone.0071110)
Supplement: Table S5 — The primers used for analysis of gene expression by qRT-PCR. (DOC) [file pone.0071110.s005.doc]

**Table 4 Gene-specific primers used for gene expression analysis by quantitative real-time PCR.**

| Unigenes | Gene | Forward primer (5' to 3') | Revers primer (5' to 3') | Product size(bp) |
| --- | --- | --- | --- | --- |
| Unigene_920 | *CHS* | ACCCTGCTAACCATGTGCTC | TGAGGTCGGTCATGTGTTCG | 81 |
| Unigene_29246 | *CHI* | CCAGGAACCTCACCACAGCAT | ATTTGGGAACCGTTAGGGATA | 157 |
| Unigene_682 | *F3H* | CTTCTTCGCTTTACCTCCTG | TCACTTTCACCCACCCTTCC | 197 |
| Unigene_396 | *F3'H* | TCAACATCGGAGATTTCGTGC | GAGTTCCACCCTCACCGTCAA | 216 |
| Unigene_28310 | *FLS* | ACTAGGAATGTGATCGCACCA | TCAGAGGGATTAGGTTTACGG | 161 |
| Unigene_7597 | *DFR* | TTCTCATCCACTCCTTCAAACG | TATTACCGCACTCTCTCCTATC | 240 |
| Unigene_7701 | *LDOX* | TGATTTCTAACGCTTACTTTGT | AAGGATGAAGTCTGAGTCTGAA | 183 |
| Unigene_16036 | *ANR* | CTCCACCATATCATCATACATCG | AAGAGTGCTTATCAAGTGCGAAA | 107 |
| Internal control | *TIPS*-*41* | TGAAGAGCAGATTGATTTGGCT | ACACTCCATTGTCAGCCAGTT | 100 |
